# Report of the Board of Health of Atlanta, 1886

**Published:** 1887-03

**Authors:** 


### REPORT OF THE BOARD OF HEALTH OF ATLANTA, 1886.

The organization of the Board is as follows:

W. S. Armstrong, M. D., President.

James B. Baird, M. D., Secretary.

James F. Alexander, M. D.

F. P. Rice, Esq.

Aaron Haas, Esq.

Sanitary Directors—W. A. King and Thomas E. Veal.

The Report makes a pamphlet of 44 octavo pages, and is neatly printed and ably gotten up. It contains many interesting items of information on sanitary matters, including the disposal of night soil and garbage; food adulteration; sewers; water supply, etc.

It is gratifying to learn that the contract made with the Newark Filtering Company, and the construction of the storm-dam recommended by the Board, promise to give the city most excellent water.

It would appear from analyses made that water taken from the reservoir and its two tributary streams, is essentially the same as that from the Chattahoochee River. The artesian water shows, also, a good quality. It is spoken of in the report as "A well located near the centre of the city. It is 2,044 feet deep, and is supplied by four veins, at 102, 225, 450, and 1,160 feet, respectively. The casing is securely plugged 1,169 feet below the surface, or 9 feet below the largest vein, by a heart-pine plug seven feet long. The vein at 1,160 feet is said to supply nine-tenths of the water."

The vital statistics for the year 1886 is very favorable, showing the remarkable health of the city, and presenting as the total annual death-rate for the whites, only 10.10, and for the colored, 21.71.

Among the principal causes of death, consumption stands charged with 119—white 49, colored 70 ; pneumonia 81—white 24, colored 57 ; other acute lung disease 25—white 9, colored 16 ; diarrhœal diseases, including diarrhœa, enteritis, dysentery and cholera infantum, 192—white 81, colored 111. Of this number, cholera infantum alone carried off 68—white 32, colored 36 ; and of this number, 42—white 21, colored 21—occurred in June, July and August, against 26 in the remaining nine months of the year. Typhoid fever 29—white 10, colored 19 ; malarial fever 6—white 1, colored 5 ; scarlet fever 3—white 1, colored 2 ; diphtheria 5—white 4, colored 1 ; rheumatism 3—white 1, colored 2.

Twenty-nine deaths were due to accident and violence—21 white and 8 colored. There were 69 still-born children reported—34 white, 35 colored.

The greatest mortality among the whites occurred in the month of June, when it reached 58 ; the lowest, in December, was 20. Among the blacks, the greatest mortality occurred in the month of June, 72 ; the lowest in November, 17.

46.74 per cent. of the deaths occurred among children under five years old. Consumption caused 13.34 per cent. of all deaths ; acute lung diseases, 11.87 per cent. ; diarrhœal diseases, 21.52 per cent., and typhoid fever, 3.25 per cent.

Consumption, acute lung diseases and diarrhœal diseases caused 46.74 per cent. of all deaths ; all other diseases, 53.25 per cent.

